# Supplementary material for: Distinct codes for environment structure and symmetry in postrhinal and retrosplenial cortices
Source: Nat Commun. 2024 Sep 13;15:8025. doi: 10.1038/s41467-024-52315-4 (PMC11399390; doi:10.1038/s41467-024-52315-4)
Supplement: Supplementary file 1 — Supplementary Information [file 41467_2024_52315_MOESM1_ESM.pdf]

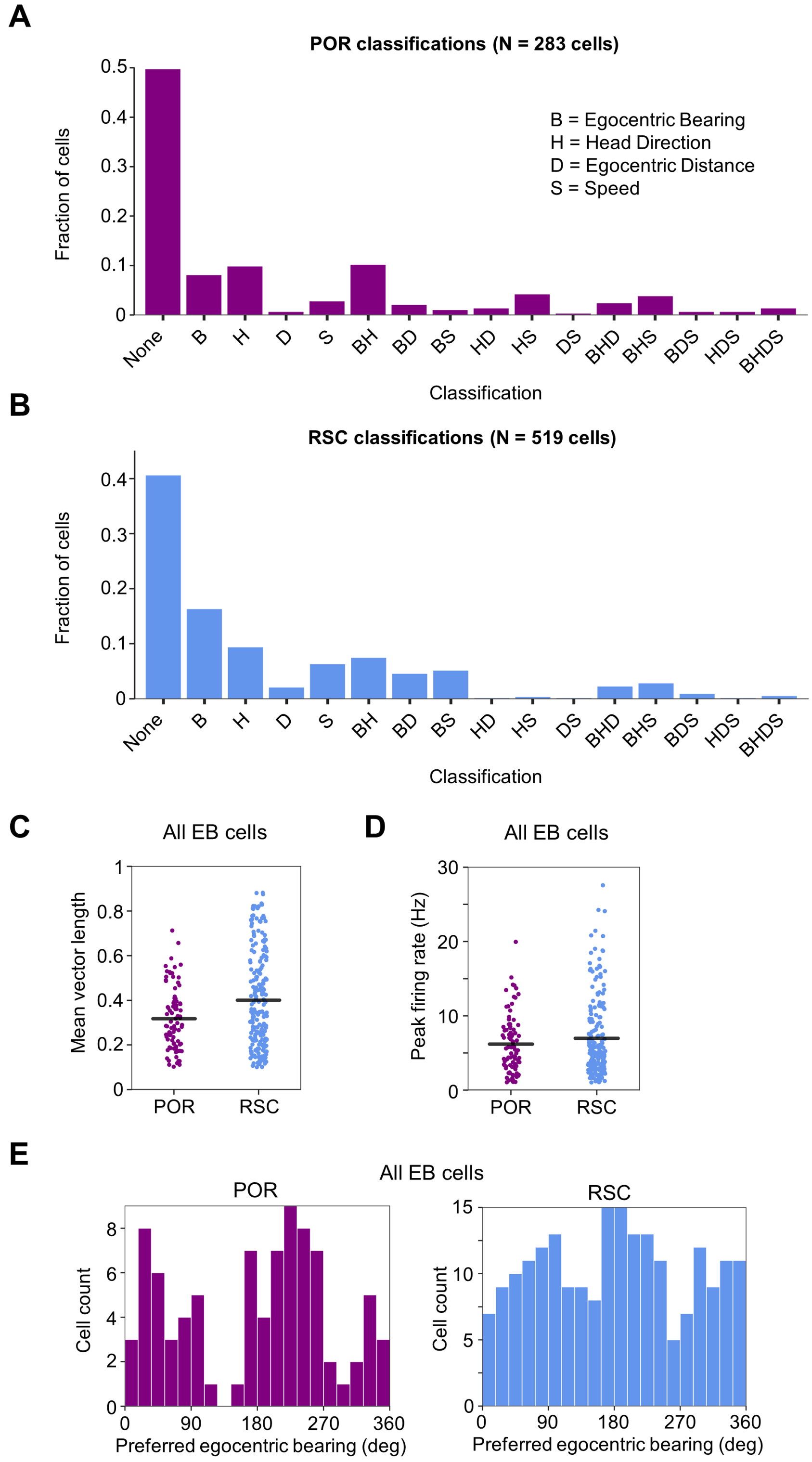

Supplementary Figure 1. Cell classifications and EB cell baseline tuning properties. A) Bar plot showing the fraction of POR cells classified as encoding each combination of behavioral variables. B) Same as (A) but for RSC cells. C) Distribution of mean vector lengths for all POR (*left*; N = 85 cells) and RSC (*right*; N = 210 cells) EB cells recorded in the square environment.

D) Same as (C) but for peak firing rates. E) Histogram of preferred egocentric bearings for all POR (*left*; N = 85 cells) and RSC (*right*; N = 210 cells) EB cells recorded in the square environment.

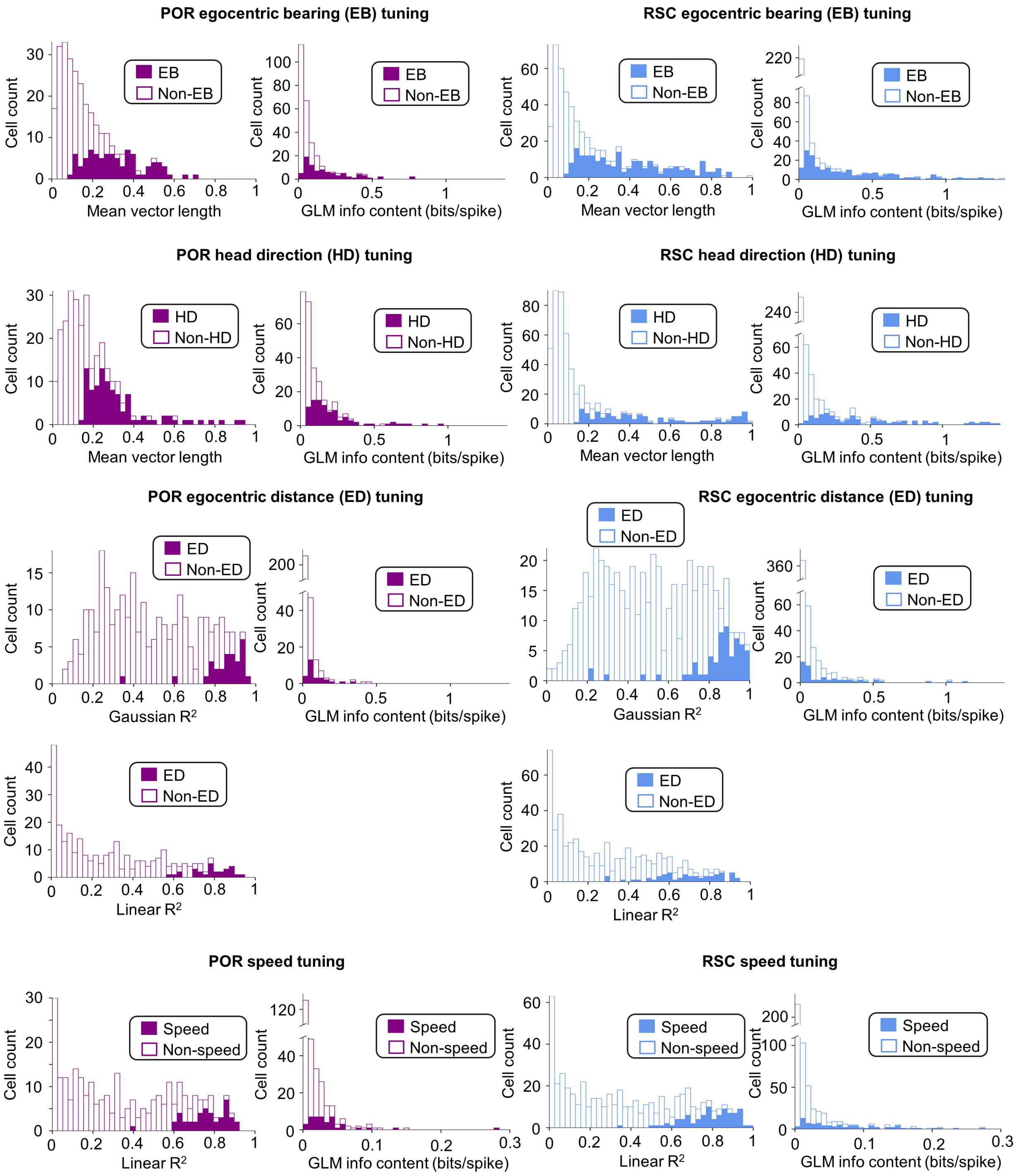

Supplementary Figure 2. Measures of tuning strength for tuned and non-tuned cells. Collection of stacked histograms showing tuning strength measures relative to each behavioral variable for POR (*left*) and RSC (*right*) cells classified as being significantly tuned to that variable (filled bars) or not (unfilled bars). Note that cells were classified based on a within-cell shuffle distribution based on one or more tuning measures as well as cross-validation with a GLM (see Methods).

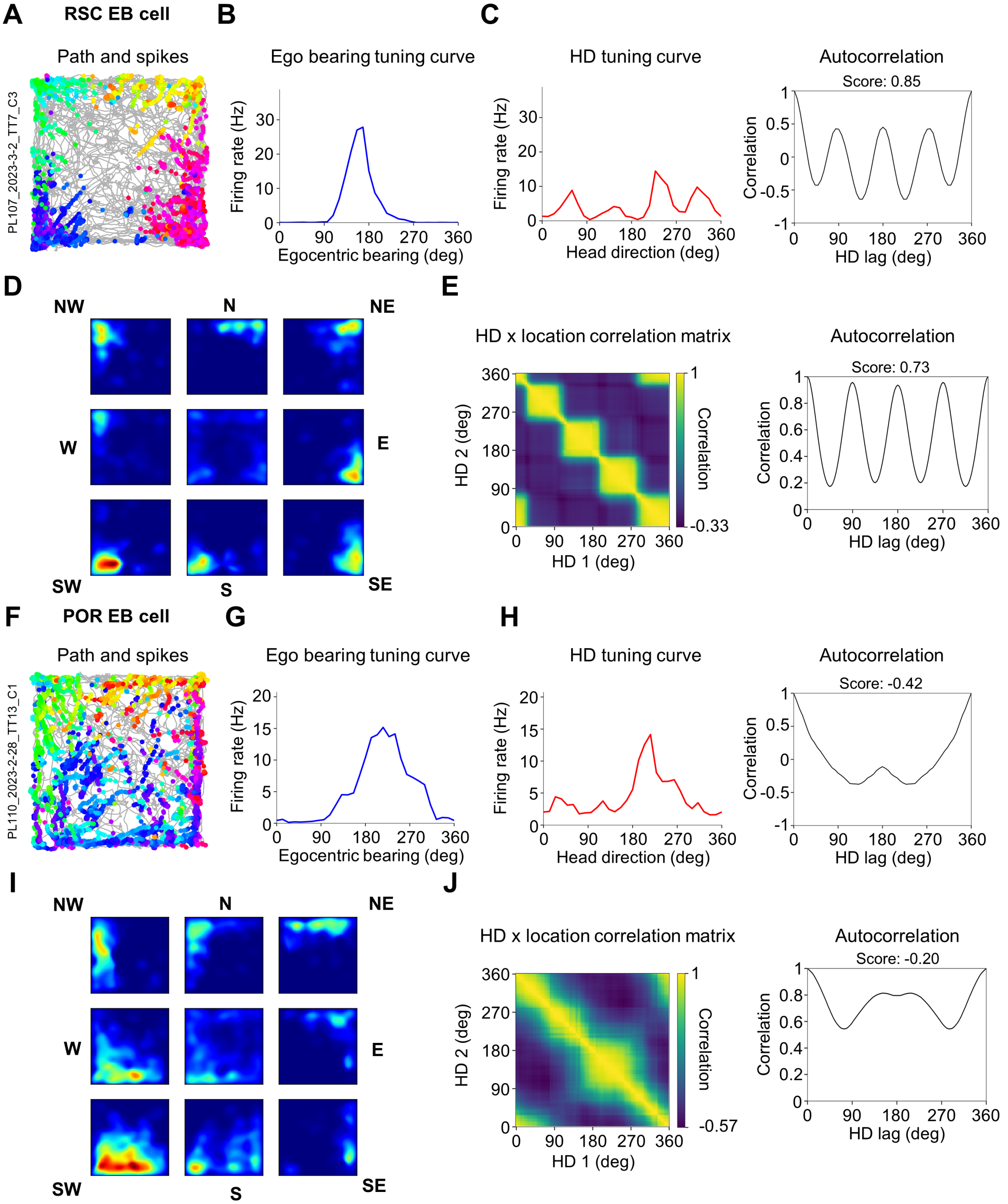

Supplementary Figure 3. Assessment of HD and HD x location symmetry. A) Directional spike plot for an RSC EB cell recorded in the square environment. B) Egocentric bearing tuning curve for the cell in (A). C) HD tuning curve (*left*) and associated autocorrelation function (*right*) for the cell in (A). D) Allocentric location firing rate maps for the cell in (A), separated according to the animal's HD (middle plot collapses across all HDs). E) HD x location correlation matrix (*left*) and associated autocorrelation function (*right*) for the cell in (A). F-J)

Same as (A-E) but for a POR EB cell. Note that, while the RSC EB cell shows strong four-fold symmetry across all domains, the POR EB cell does not.

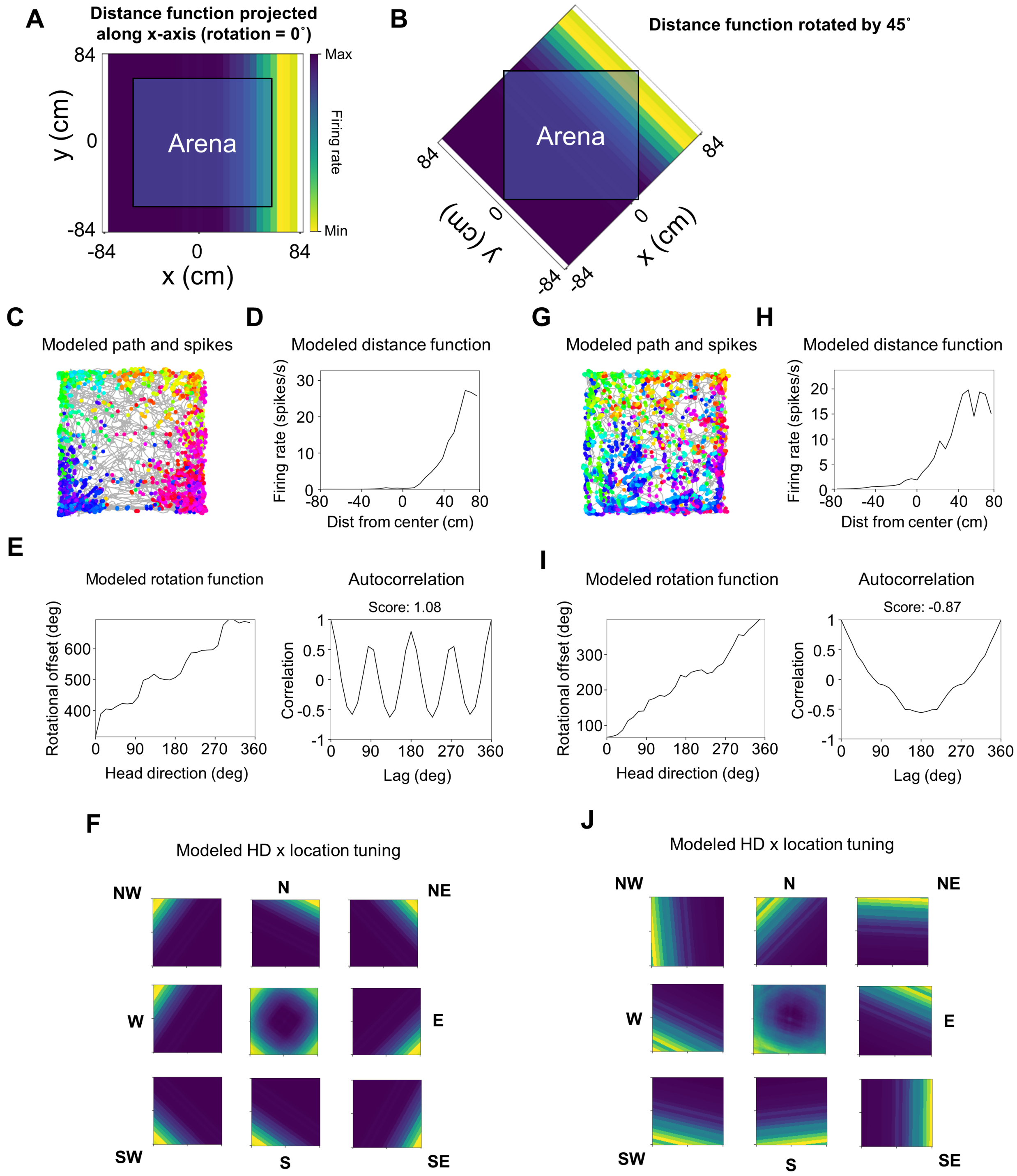

Supplementary Figure 4. GLM analysis of radial symmetry. A) Example 2D projection of a 1D distance function across the environment with a rotation of 0°. B) Same 2D projection as (A) but rotated by 45° with respect to the environment. C) Simulated directional spike plot for an RSC EB cell (same as Supplementary Fig. 3A) modeled using the GLM radial symmetry analysis. D) GLM-derived distance function for the cell in (C). E) GLM-derived rotation function (*left*) and associated autocorrelation function (*right*) for the cell in (C). Simulated allocentric location firing rate maps for the cell in (C), separated according to the animal's HD

(middle plot collapses across all HDs). G-J) Same as (C-F) but for a POR EB cell (same cell as Supplementary Fig. 3F). Note the lack of four-fold symmetry in the rotation function compared to the RSC EB cell.

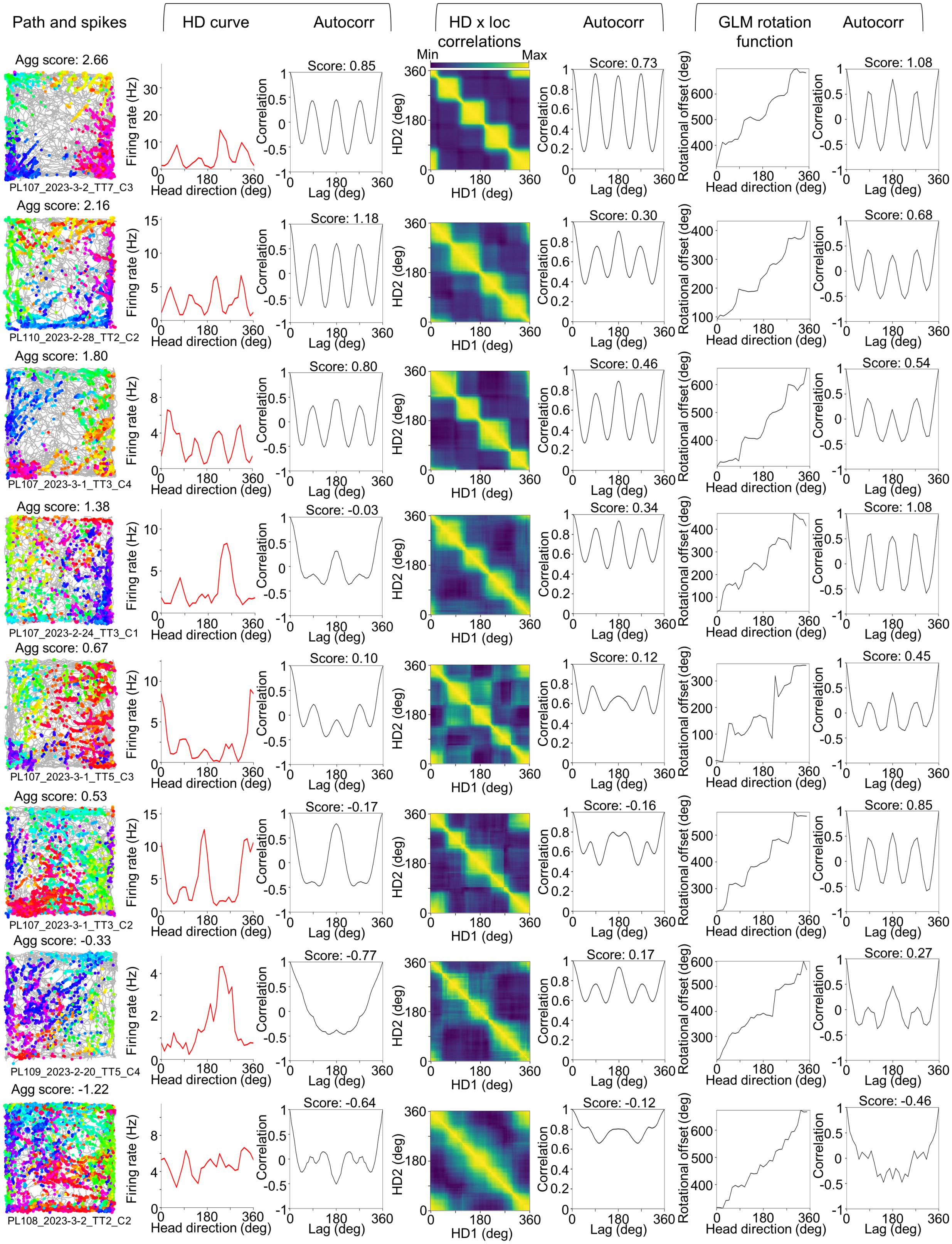
Path and spikes
HD curve
Autocorr
HD x loc correlations
Autocorr
GLM rotation function
Autocorr
Min
Max
Agg score: 2.66
PL107_2023-3-2_TT7_C3
Score: 0.85
Score: 0.73
Score: 1.08
Agg score: 2.16
PL110_2023-2-28_TT2_C2
Score: 1.18
Score: 0.30
Score: 0.68
Agg score: 1.80
PL107_2023-3-1_TT3_C4
Score: 0.80
Score: 0.46
Score: 0.54
Agg score: 1.38
PL107_2023-2-24_TT3_C1
Score: -0.03
Score: 0.34
Score: 1.08
Agg score: 0.67
PL107_2023-3-1_TT5_C3
Score: 0.10
Score: 0.12
Score: 0.45
Agg score: 0.53
PL107_2023-3-1_TT3_C2
Score: -0.17
Score: -0.16
Score: 0.85
Agg score: -0.33
PL109_2023-2-20_TT5_C4
Score: -0.77
Score: 0.17
Score: 0.27
Agg score: -1.22
PL108_2023-3-2_TT2_C2
Score: -0.64
Score: -0.12
Score: -0.46
Firing rate (Hz)
Head direction (deg)
Correlation
Lag (deg)
HD2 (deg)
HD1 (deg)
Rotational offset (deg)

Supplementary Figure 5. Symmetry analyses for example RSC EB cells. Directional spike plots, HD tuning curves, HD x location correlation matrices, and GLM-derived rotation functions (along with all associated autocorrelation functions) for ten example RSC EB cells recorded in the square environment. Example cells were selected to illustrate a variety of directional preferences, with cells ordered from highest (*top*) to lowest (*bottom*) four-fold symmetry scores.

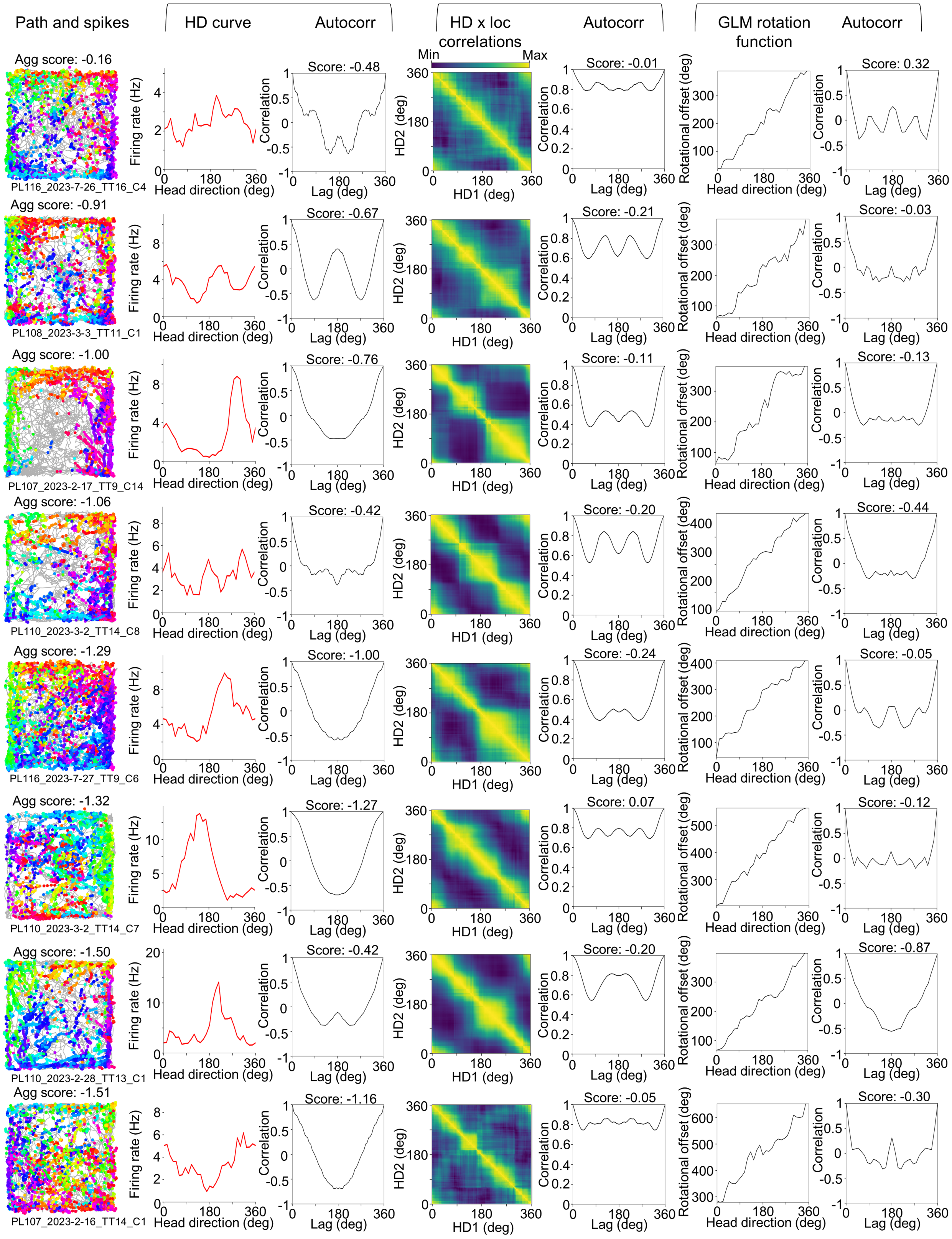

Path and spikes
HD curve
Autocorr
HD x loc correlations
Autocorr
GLM rotation function
Autocorr
Min
Max
Agg score: -0.16
PL116_2023-7-26_TT16_C4
Score: -0.48
Score: -0.01
Score: 0.32
Agg score: -0.91
PL108_2023-3-3_TT11_C1
Score: -0.67
Score: -0.21
Score: -0.03
Agg score: -1.00
PL107_2023-2-17_TT9_C14
Score: -0.76
Score: -0.11
Score: -0.13
Agg score: -1.06
PL110_2023-3-2_TT14_C8
Score: -0.42
Score: -0.20
Score: -0.44
Agg score: -1.29
PL116_2023-7-27_TT9_C6
Score: -1.00
Score: -0.24
Score: -0.05
Agg score: -1.32
PL110_2023-3-2_TT14_C7
Score: -1.27
Score: 0.07
Score: -0.12
Agg score: -1.50
PL110_2023-2-28_TT13_C1
Score: -0.42
Score: -0.20
Score: -0.87
Agg score: -1.51
PL107_2023-2-16_TT14_C1
Score: -1.16
Score: -0.05
Score: -0.30
Firing rate (Hz)
Head direction (deg)
Correlation
Lag (deg)
HD2 (deg)
HD1 (deg)
Rotational offset (deg)

Supplementary Figure 6. Symmetry analyses for example POR EB cells. Directional spike plots, HD tuning curves, HD x location correlation matrices, and GLM-derived rotation functions (along with all associated autocorrelation functions) for ten example POR EB cells recorded in the square environment. Example cells were selected to illustrate a variety of directional preferences, with cells ordered from highest (*top*) to lowest (*bottom*) four-fold symmetry scores.

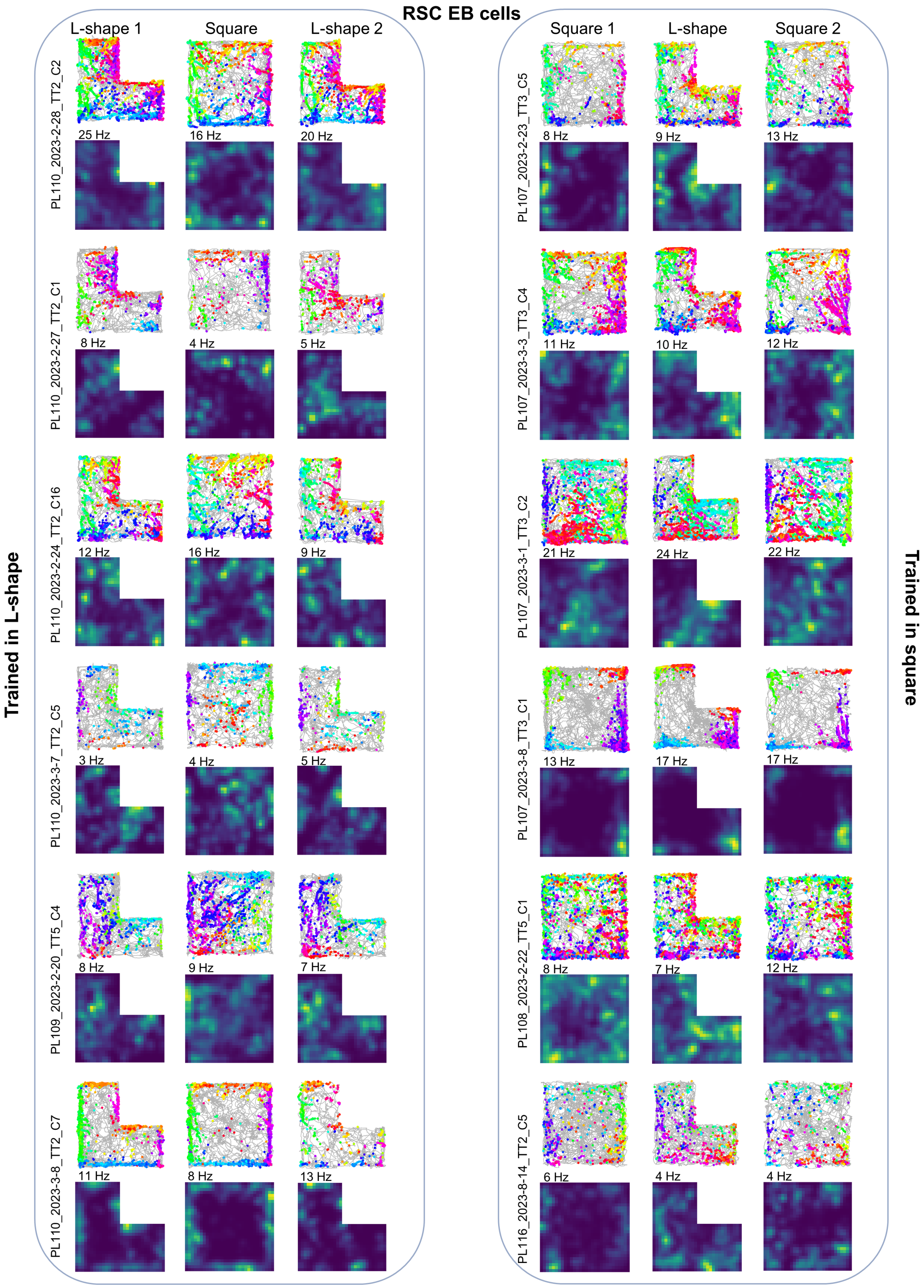
RSC EB cells
Trained in L-shape
L-shape 1
Square
L-shape 2
PL110_2023-2-28_TT2_C2
25 Hz
16 Hz
20 Hz
PL110_2023-2-27_TT2_C1
8 Hz
4 Hz
5 Hz
PL110_2023-2-24_TT2_C16
12 Hz
16 Hz
9 Hz
PL110_2023-3-7_TT2_C5
3 Hz
4 Hz
5 Hz
PL109_2023-2-20_TT5_C4
8 Hz
9 Hz
7 Hz
PL110_2023-3-8_TT2_C7
11 Hz
8 Hz
13 Hz
Trained in square
Square 1
L-shape
Square 2
PL107_2023-2-23_TT3_C5
8 Hz
9 Hz
13 Hz
PL107_2023-3-3_TT3_C4
11 Hz
10 Hz
12 Hz
PL107_2023-3-1_TT3_C2
21 Hz
24 Hz
22 Hz
PL107_2023-3-8_TT3_C1
13 Hz
17 Hz
17 Hz
PL108_2023-2-22_TT5_C1
8 Hz
7 Hz
12 Hz
PL116_2023-8-14_TT2_C5
6 Hz
4 Hz
4 Hz

Supplementary Figure 7. Example RSC EB cells in the L-shape experiment. Directional spike plots and allocentric location firing rate maps for twelve example RSC EB cells recorded in the L-shape experiment, six from animals trained in the L-shape (*left*) and six from animals trained in the square (*right*).

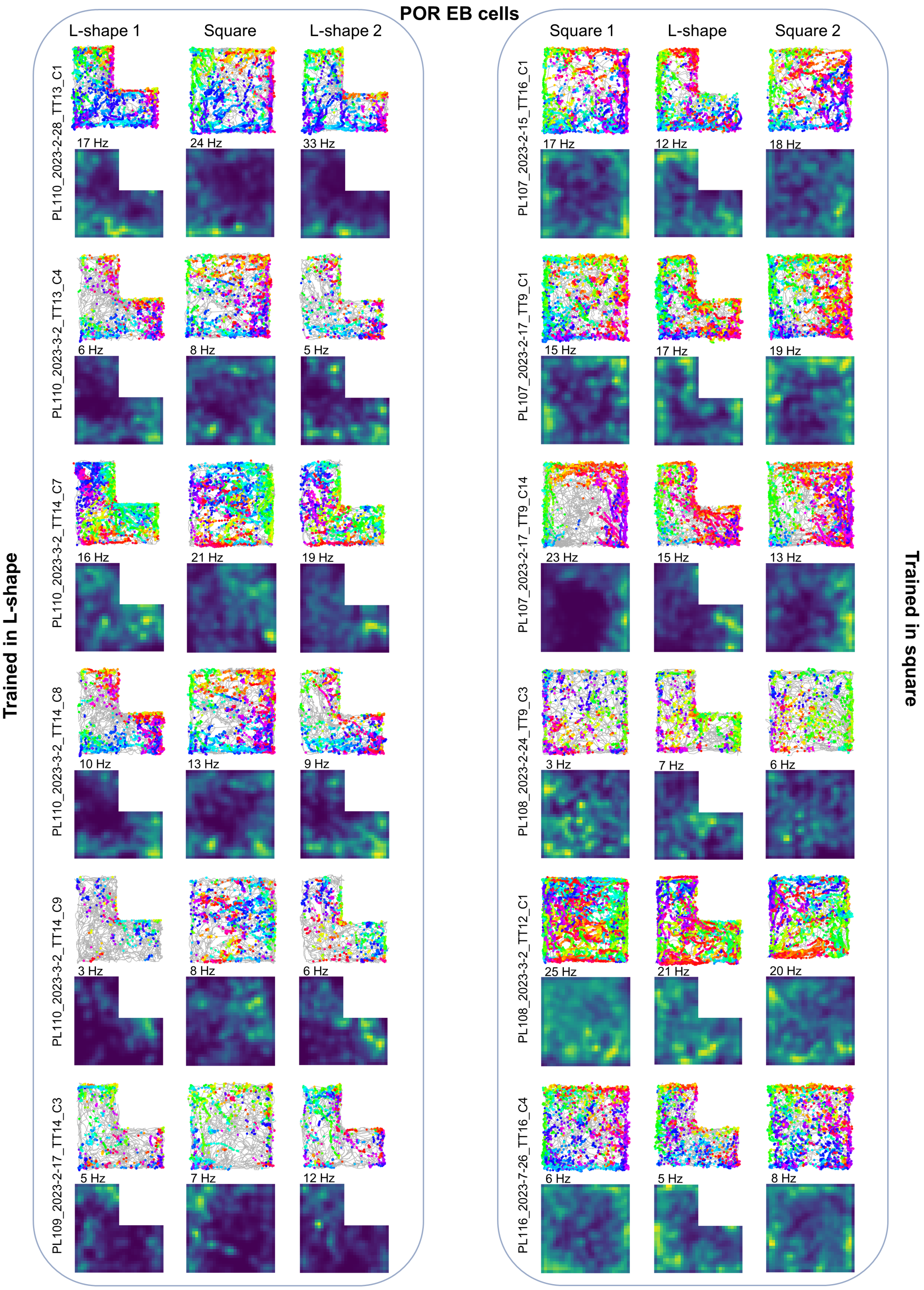
POR EB cells
Trained in L-shape
L-shape 1
Square
L-shape 2
PL110_2023-2-28_TT13_C1
17 Hz
24 Hz
33 Hz
PL110_2023-3-2_TT13_C4
6 Hz
8 Hz
5 Hz
PL110_2023-3-2_TT14_C7
16 Hz
21 Hz
19 Hz
PL110_2023-3-2_TT14_C8
10 Hz
13 Hz
9 Hz
PL110_2023-3-2_TT14_C9
3 Hz
8 Hz
6 Hz
PL109_2023-2-17_TT14_C3
5 Hz
7 Hz
12 Hz
Trained in square
Square 1
L-shape
Square 2
PL107_2023-2-15_TT16_C1
17 Hz
12 Hz
18 Hz
PL107_2023-2-17_TT9_C1
15 Hz
17 Hz
19 Hz
PL107_2023-2-17_TT9_C14
23 Hz
15 Hz
13 Hz
PL108_2023-2-24_TT9_C3
3 Hz
7 Hz
6 Hz
PL108_2023-3-2_TT12_C1
25 Hz
21 Hz
20 Hz
PL116_2023-7-26_TT16_C4
6 Hz
5 Hz
8 Hz

Supplementary Figure 8. Example POR EB cells in the L-shape experiment. Directional spike plots and allocentric location firing rate maps for twelve example POR EB cells recorded in the L-shape experiment, six from animals trained in the L-shape (*left*) and six from animals trained in the square (*right*).

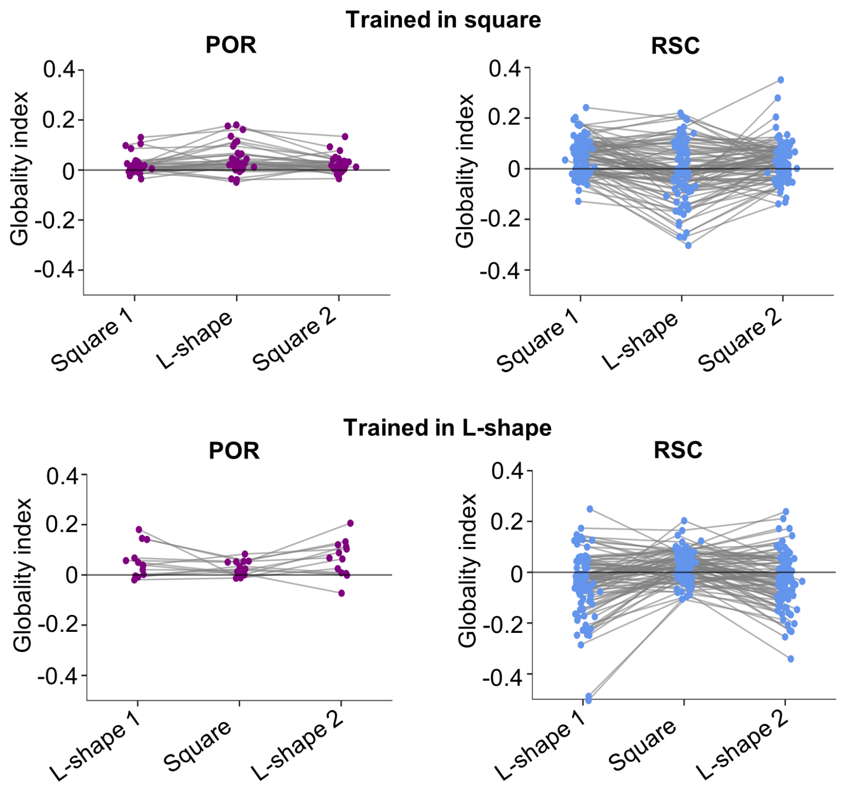

Supplementary Figure 9. Local vs. global coding and habituation. Globality indices derived from the local vs. global GLM analysis for all POR EB cells (*left*) and all RSC EB cells (*right*) recorded across all three sessions of the L-shape experiment, for animals initially trained in the square environment (*top*; POR: N = 33 cells, RSC: N = 89 cells) and those trained in the L-shaped environment (*bottom*; POR: N = 13 cells, RSC: N = 88 cells).

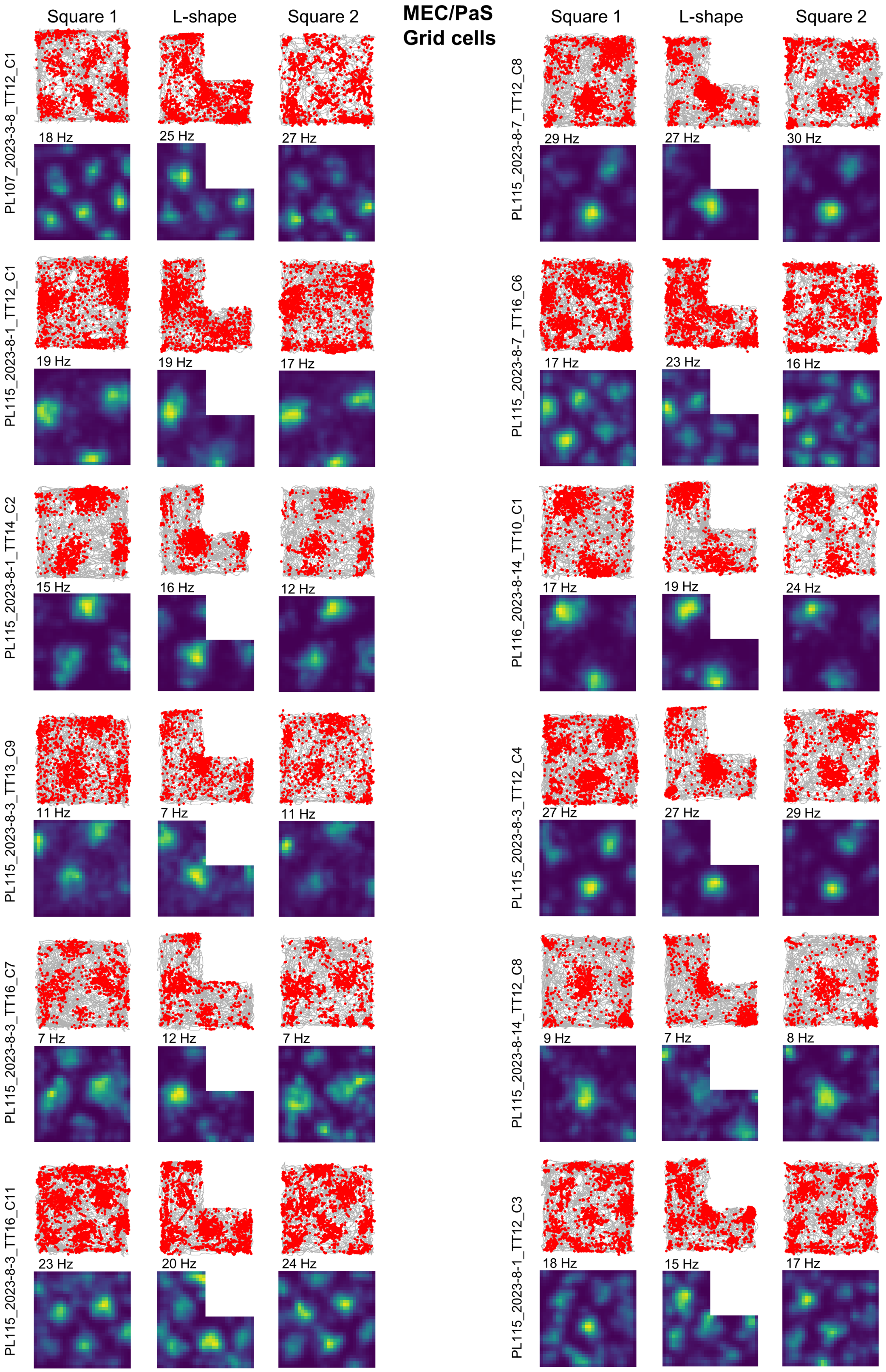

MEC/PaS
Grid cells
Square 1
L-shape
Square 2
Square 1
L-shape
Square 2
PL107_2023-3-8_TT12_C1
18 Hz
25 Hz
27 Hz
PL115_2023-8-7_TT12_C8
29 Hz
27 Hz
30 Hz
PL115_2023-8-1_TT12_C1
19 Hz
19 Hz
17 Hz
PL115_2023-8-7_TT16_C6
17 Hz
23 Hz
16 Hz
PL115_2023-8-1_TT14_C2
15 Hz
16 Hz
12 Hz
PL116_2023-8-14_TT10_C1
17 Hz
19 Hz
24 Hz
PL115_2023-8-3_TT13_C9
11 Hz
7 Hz
11 Hz
PL115_2023-8-3_TT12_C4
27 Hz
27 Hz
29 Hz
PL115_2023-8-3_TT16_C7
7 Hz
12 Hz
7 Hz
PL115_2023-8-14_TT12_C8
9 Hz
7 Hz
8 Hz
PL115_2023-8-3_TT16_C11
23 Hz
20 Hz
24 Hz
PL115_2023-8-1_TT12_C3
18 Hz
15 Hz
17 Hz

Supplementary Figure 10. Grid cells in the L-shape experiment. Path and spike plots and allocentric location firing rate maps for twelve example MEC/PaS grid cells recorded across all three sessions of the L-shape experiment (all grid cells came from animals trained in the square environment).

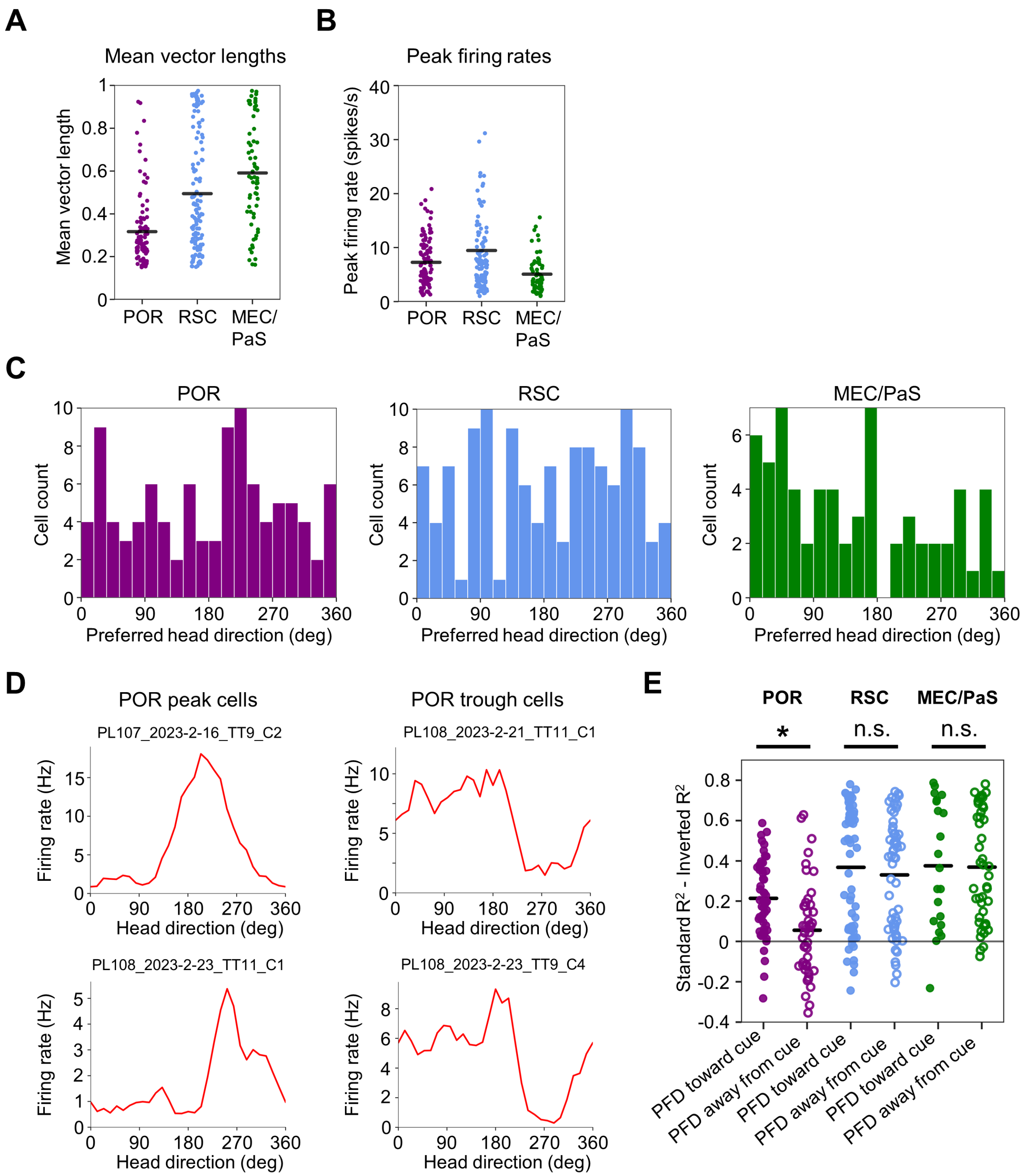

Supplementary Figure 11. HD cell baseline firing properties. A) Mean vector lengths for all POR (*left*; N = 97 cells), RSC (*middle*; N = 122 cells), and MEC/PaS (*right*; N = 65 cells) HD cells recorded in the square environment. B) Same as (A) but for peak firing rates. C) Histogram of preferred HDs for all POR (*left*), RSC (*middle*), and MEC/PaS (*right*) HD cells recorded in the square environment. D) Tuning curves for two example POR peak cells (*left*) and two example POR trough cells (*right*) recorded in the square environment. E) Difference between standard and inverted von Mises R^2 values for HD cells in POR, RSC, and MEC/PaS, separated into HD cells with maximal firing rates oriented toward or away from the familiar landmark cue. Note

that only POR shows a significant distinction between these two types of HD cells (POR toward vs. away, two-sided Wilcoxon rank sum test, $Z = 3.66$, $P = 2.52e\text{-}4$; all other comparisons $P > 0.05$).

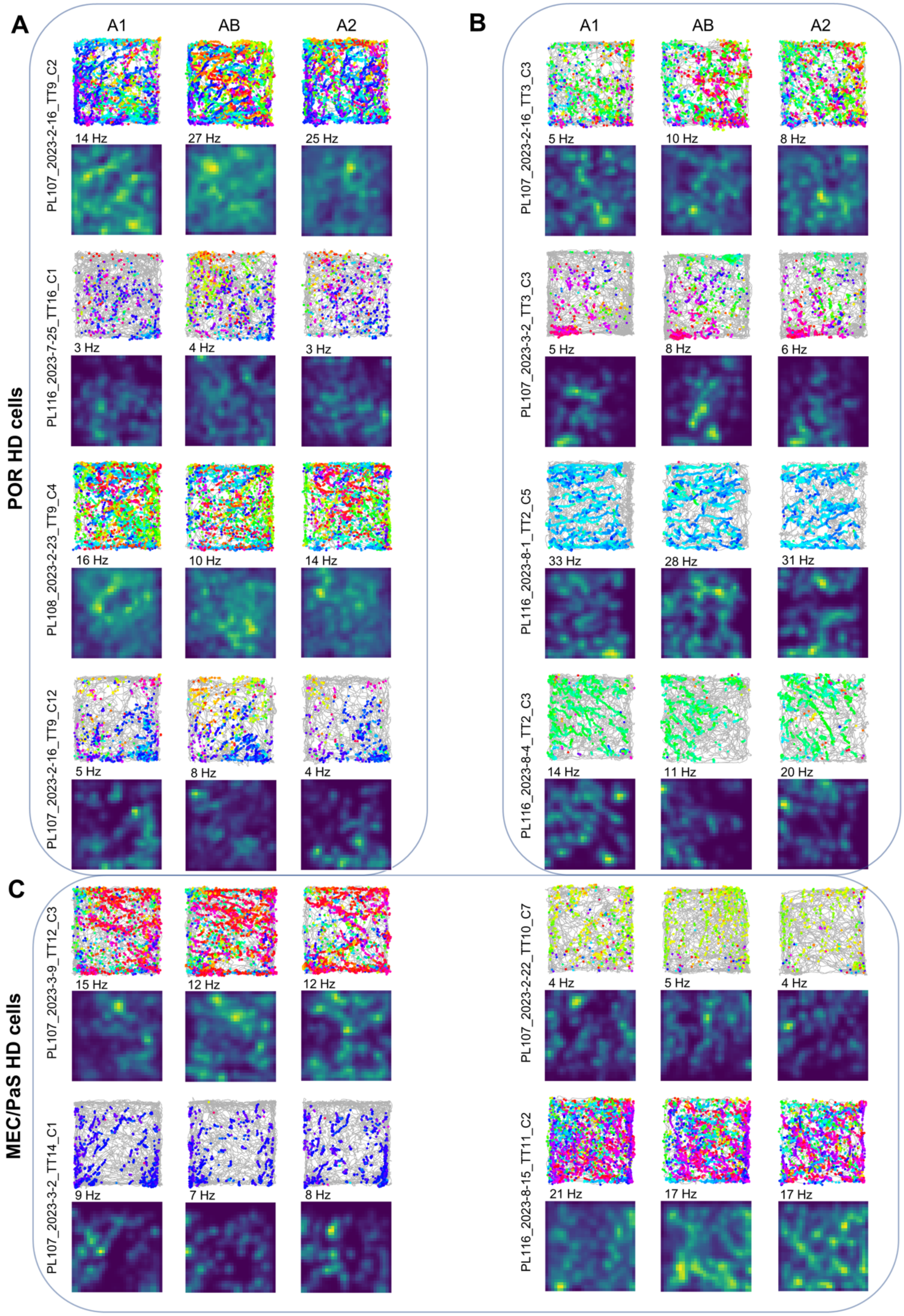

A
B
C
A1
AB
A2
POR HD cells
RSC HD cells
MEC/PaS HD cells
MEC/PaS HD cells
PL107_2023-2-16_TT9_C2
14 Hz
27 Hz
25 Hz
PL116_2023-7-25_TT16_C1
3 Hz
4 Hz
3 Hz
PL108_2023-2-23_TT9_C4
16 Hz
10 Hz
14 Hz
PL107_2023-2-16_TT9_C12
5 Hz
8 Hz
4 Hz
PL107_2023-2-16_TT3_C3
5 Hz
10 Hz
8 Hz
PL107_2023-3-2_TT3_C3
5 Hz
8 Hz
6 Hz
PL116_2023-8-1_TT2_C5
33 Hz
28 Hz
31 Hz
PL116_2023-8-4_TT2_C3
14 Hz
11 Hz
20 Hz
PL107_2023-3-9_TT12_C3
15 Hz
12 Hz
12 Hz
PL107_2023-3-2_TT14_C1
9 Hz
7 Hz
8 Hz
PL107_2023-2-22_TT10_C7
4 Hz
5 Hz
4 Hz
PL116_2023-8-15_TT11_C2
21 Hz
17 Hz
17 Hz

Supplementary Figure 12. Location firing properties of HD cells in the AB experiment. A) Directional spike plots and allocentric location firing rate maps for the four POR HD cells with tuning curves shown in Fig. 5A. B) Same as (A) but for the four RSC HD cells from Fig. 5B. C) Same as (A) but for the four MEC/PaS cells from Fig. 5C. Note that the POR HD cells and the top two RSC HD cells tend to show more bimodal distributions of HD colors in the AB condition.

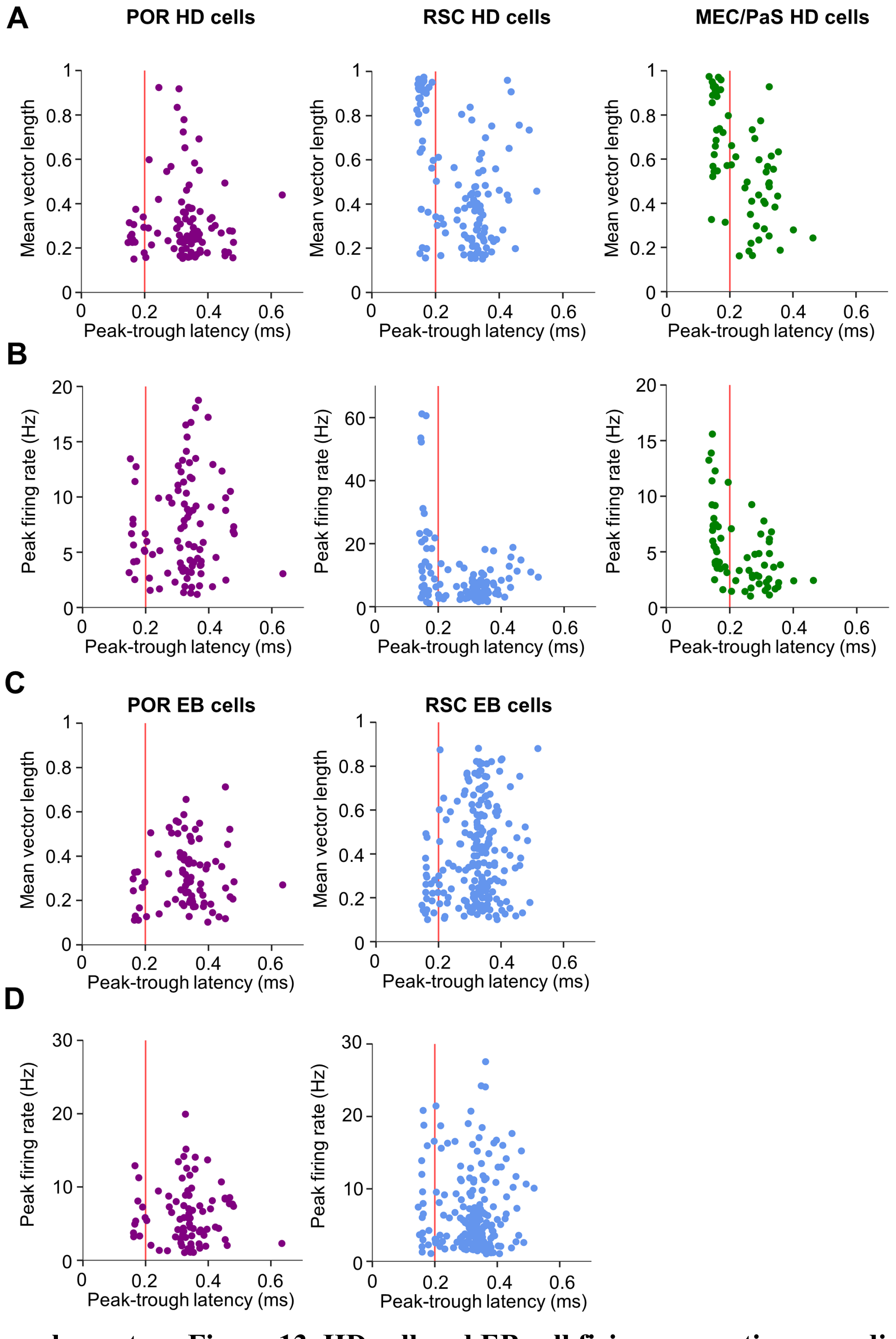

Supplementary Figure 13. HD cell and EB cell firing properties according to waveform width. A) Comparison between HD mean vector length and extracellular waveform width for all HD cells recorded in the square environment from POR (*left*; N = 97 cells), RSC (*middle*; N = 122 cells), and MEC/PaS (*right*; N = 65 cells). MVLs were higher for narrow-waveform HD cells in RSC (two-sided Wilcoxon rank sums test, $Z = 5.87$, $P = 4.29e\text{-}9$) and MEC ($Z = 5.05$, $P = 4.32e\text{-}7$) but not POR ($P > 0.05$) B) Same as (A) but for peak firing rates. Note that RSC (two-sided Wilcoxon rank sums test, $Z = 4.16$, $P = 3.22e\text{-}5$) and MEC/PaS ($Z = 4.26$, $P = 2.02e\text{-}5$) HD

cells with narrow waveforms tend to have higher mean vector lengths and peak firing rates (POR: $P > 0.05$). C) Comparison between egocentric mean vector length and extracellular waveform width for all EB cells recorded in the square environment from POR (*left*; N = 85 cells) and RSC (*right*; N = 210 cells). D) Same as (C) but for peak firing rates. Note the lack of strong distinction between narrow and broad waveform EB cells in terms of mean vector lengths or peak firing rates, unlike HD cells.

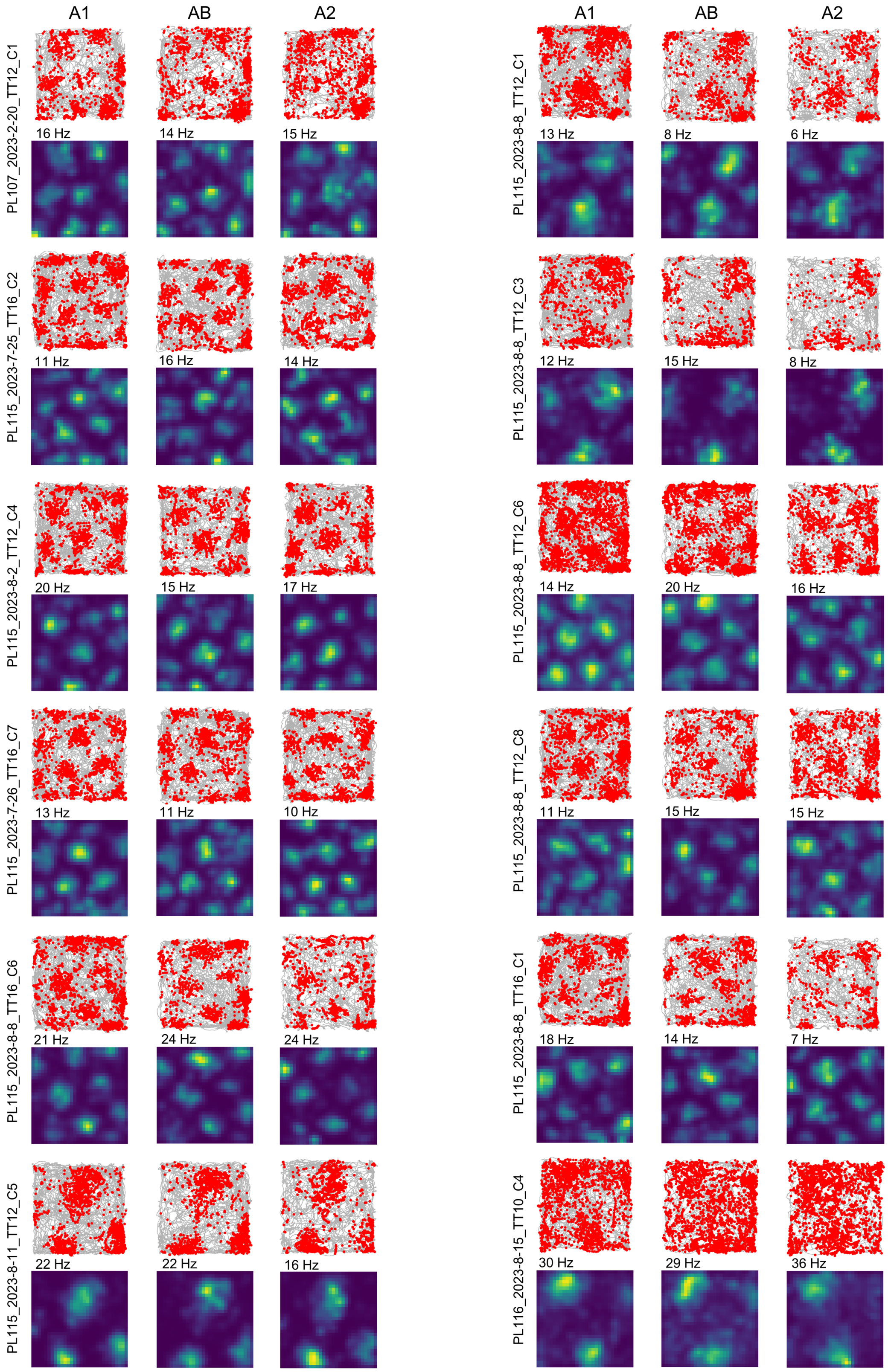
A1
AB
A2
PL107_2023-2-20_TT12_C1
16 Hz
14 Hz
15 Hz
PL115_2023-7-25_TT16_C2
11 Hz
16 Hz
14 Hz
PL115_2023-8-2_TT12_C4
20 Hz
15 Hz
17 Hz
PL115_2023-7-26_TT16_C7
13 Hz
11 Hz
10 Hz
PL115_2023-8-8_TT16_C6
21 Hz
24 Hz
24 Hz
PL115_2023-8-11_TT12_C5
22 Hz
22 Hz
16 Hz
A1
AB
A2
PL115_2023-8-8_TT12_C1
13 Hz
8 Hz
6 Hz
PL115_2023-8-8_TT12_C3
12 Hz
15 Hz
8 Hz
PL115_2023-8-8_TT12_C6
14 Hz
20 Hz
16 Hz
PL115_2023-8-8_TT12_C8
11 Hz
15 Hz
15 Hz
PL115_2023-8-8_TT16_C1
18 Hz
14 Hz
7 Hz
PL116_2023-8-15_TT10_C4
30 Hz
29 Hz
36 Hz

Supplementary Figure 14. Grid cells in the AB experiment. Path and spike plots and allocentric location firing rate maps for twelve example MEC/PaS grid cells recorded across the three sessions of the AB experiment. Note the strong consistency of grid cell firing patterns across sessions (N = 61 grid cells; A1-A2 rate map correlations vs. A1-AB correlations, two-sided Wilcoxon signed-rank test, $W = 879$, $P = 0.63$).

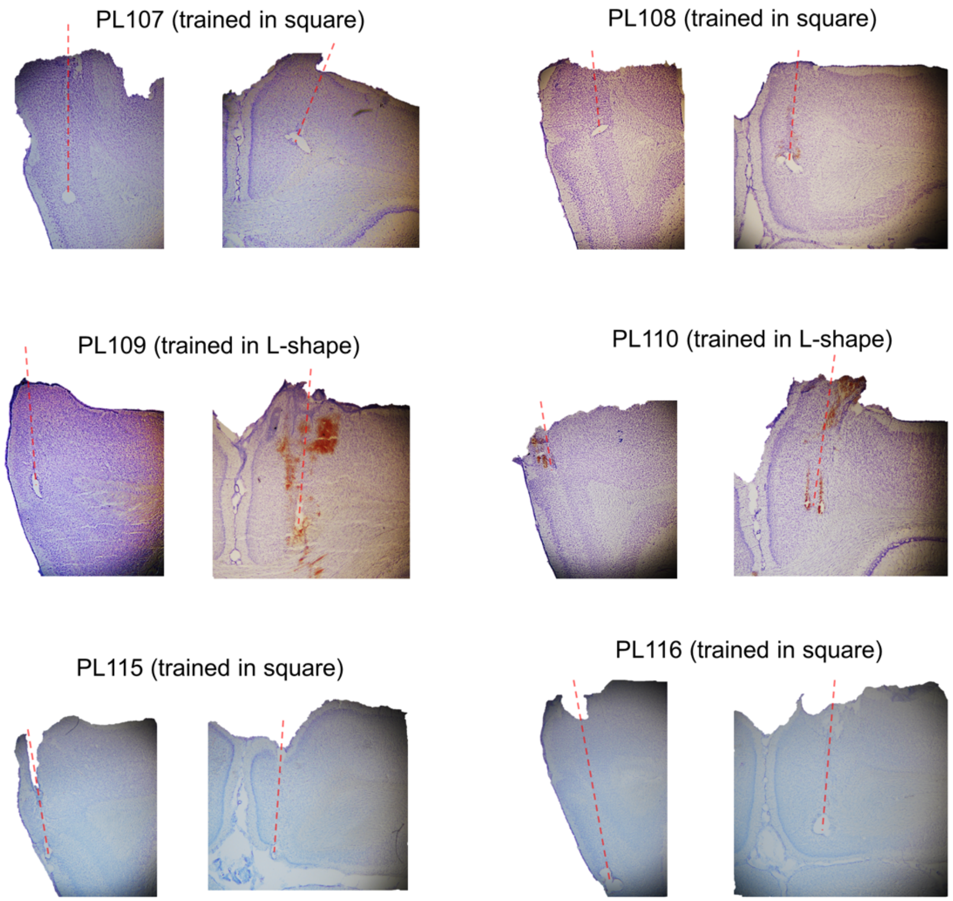

Supplementary Figure 15. Histology. Nissl-stained sagittal (*left*) and coronal (*right*) sections for all animals showing electrode tracks through regions of interest. Electrode tracks are indicated by a red dotted line.
